# Sparsely methylated mitochondrial cell free DNA released from cardiomyocytes contributes to systemic inflammatory response accompanied by atrial fibrillation

**DOI:** 10.1038/s41598-021-85204-7

**Published:** 2021-03-18

**Authors:** Masahiro Yamazoe, Tetsuo Sasano, Kensuke Ihara, Kentaro Takahashi, Wakana Nakamura, Naomi Takahashi, Hiroaki Komuro, Satomi Hamada, Tetsushi Furukawa

**Affiliations:** 1 Department of Bio-Informational Pharmacology, Medical Research Institute, Tokyo Medical and Dental University (TMDU), Tokyo, Japan; 2 Department of Cardiovascular Physiology, Tokyo Medical and Dental University (TMDU), Tokyo, Japan; 3 Department of Cardiovascular Medicine, Tokyo Medical and Dental University (TMDU), 1-5-45 Yushima, Bunkyo-ku, Tokyo, 113-8519 Japan

**Keywords:** Biomarkers, Cardiology, Pathogenesis

## Abstract

Systemic inflammation is assumed to be the consequence and the cause of atrial fibrillation (AF); however, the underlying mechanism remains unclear. We aimed to evaluate the level of cell-free DNA (cfDNA) in patients with AF and AF mimicking models, and to illuminate its impact on inflammation. Peripheral blood was obtained from 54 patients with AF and 104 non-AF controls, and cfDNA was extracted. We extracted total cfDNA from conditioned medium after rapid pacing to HL-1 cells. Nuclear and mitochondrial DNA were separately extracted and fragmented to simulate nuclear-cfDNA (n-cfDNA) and mitochondrial-cfDNA (mt-cfDNA). The AF group showed higher cfDNA concentration than the non-AF group (12.6 [9.0–17.1] vs. 8.1 [5.3–10.8] [ng/mL], *p* < 0.001). The copy numbers of n-cfDNA and mt-cfDNA were higher in AF groups than in non-AF groups; the difference of mt-cfDNA was particularly apparent (*p* = 0.011 and *p* < 0.001, respectively). Administration of total cfDNA and mt-cfDNA to macrophages significantly promoted IL-1β and IL-6 expression through TLR9, whereas n-cfDNA did not. Induction of cytokine expression by methylated mt-cfDNA was lower than that by unmethylated mt-cfDNA. Collectively, AF was associated with an increased cfDNA level, especially mt-cfDNA. Sparsely methylated mt-cfDNA released from cardiomyocytes may be involved in sterile systemic inflammation accompanied by AF.

## Introduction

Atrial fibrillation (AF) is the most common sustained cardiac arrhythmia, and a major public health problem with increased mortality risk, a great number of comorbidities, including cerebrovascular stroke, heart failure, renal failure, and endothelial dysfunction^1,2^. Various inflammatory markers and mediators including C-reactive protein (CRP) and interleikin-6 (IL-6) have been reported to be linked with the presence and outcome of AF^3,4^. It has been reported that these systemic inflammatory responses cause AF-related systemic dysfunctions. In addition, accumulated evidence has indicated that atrial inflammation evokes AF itself via electrical and structural remodeling (AF begets AF)^5,6^. However, the underlying mechanism on how AF induces systemic inflammation remains to be elucidated.

Recently, cell-free DNA (cfDNA), which is released from cells and circulates in blood, has been recognized as a useful biomarker for diagnosis of cancer, called liquid biopsy^7^. The sequencing of cfDNA in maternal circulating blood has also been utilized for prenatal genetic diagnosis^8^. In addition, cfDNA has been attributed to damage associated molecular patterns (DAMPs) in several inflammatory disorders, such as sepsis^9^, end-stage renal dysfunction requiring hemodialysis^10^, and myocardial infarction^11^. DAMPs are recognized by pattern recognition receptors, of which Toll-like receptors (TLRs) have been most thoroughly described; especially TLR9, which is found intracellularly in endolysosomes and recognizes CpG DNA^12^ as an innate immunity mediator.

Based on these findings, we focused on cfDNA as a potential contributor for systemic inflammation accompanied by AF. In this study, we aimed to evaluate cfDNA levels both in patients with AF in clinical settings and in an AF-mimicking murine model, in order to clarify its impact on systemic inflammation. Especially, we focused on the differential implication of nuclear cfDNA (n-cfDNA) and mitochondrial cfDNA (mt-cfDNA).

## Methods

### Study subjects

One hundred four subjects without AF (non-AF) and 54 patients with AF were enrolled in this study. Non-AF group was further divided into two subgroups: 28 young healthy controls (YC) and 76 controls whose age matched those of the AF group (AC). The AF group comprised 26 paroxysmal AF patients (PAF) and 28 persistent AF patients (PerAF). Subjects in AC and AF groups were recruited from outpatients in Tokyo Medical and Dental University Medical Hospital, Tokyo, Japan. Exclusion criteria were the following: patients with cancer, myocardial infarction, hemodialysis, or inflammatory diseases. The study was approved by the ethics committee of Tokyo Medical and Dental University (No. M2000-188) and performed in accordance with these guidelines and regulations. After written informed consent was obtained from all participants, blood samples were collected from the cubital vein.

### Atrial tachycardia model in vitro and in vivo

To investigate whether cfDNA is released from cardiomyocytes under tachycardia stress, we administered rapid field stimulation to HL-1, murine atrial cardiomyocytes, using a C-Pace culture pacer (IonOptix Corporation, Amsterdam, Netherlands). HL-1 cells were kindly provided by Dr. William Claycomb (Louisiana State University Health Science Center, New Orleans, LA, USA)^13^. The pacing amplitude was set at 10 V with a 5 ms pulse width, and a 10 Hz frequency. After pacing, conditioned medium was collected. As a control of non-excitatory cell, we applied same field stimulation to NIH-3T3, murine fibroblast cells. The ratio of cell death after 24 h pacing was analyzed by propidium iodide (PI) staining with flow cytometer (SA-3800, Sony, Tokyo, Japan) according to manufacturer’s instruction.

All animal experiments were approved by Institutional Animal Care and Use Committee of Tokyo Medical and Dental University. All methods were carried out in accordance with relevant guidelines and regulations and the study was carried out in compliance with the ARRIVE guidelines. C57BL/6 male mice at the age of 8–10 weeks were purchased from CLEA Japan (Tokyo, Japan). A custom-made 1-French quadripolar catheter (Unique Medical, Tokyo, Japan) was inserted through the right jugular vein using a cut-down approach. Right atrial stimulation at a frequency of 1500 beats/min was performed using a programmable stimulator (Fukuda-Denshi, Tokyo, Japan). After 2 h of rapid pacing, blood samples were collected from the animals and stored in tubes containing EDTA-2K. For the sham operation group, mice underwent an identical surgical procedure without electrical stimulation.

### Extraction and quantification of cfDNA

cfDNA was extracted from 600 µL of conditioned medium, 600 µL of human plasma, and 100 µL of murine plasma using MagMAX Cell-Free DNA Isolation Kit (Thermo Fisher Scientific, Carlsbad, CA, USA). The concentration of cfDNA was measured using Qubit dsDNA HS Assay Kit with Qubit 4.0 Fluorometer (Thermo Fisher Scientific).

To compare the levels of n-cfDNA and mt-cfDNA in total cfDNA, the absolute copy number of those was measured using quantitative polymerase chain reaction (qPCR) targeting glyceraldehyde-3-phosphate dehydrogenase (GAPDH), and NADH dehydrogenase (NADH), respectively. The copy number was calculated using the standard curves for cloned GAPDH and NADH in a pGEM-T Easy vector (Promega, Madison, WI, USA), respectively. Primer sequences are shown in the supplementary Table S1. In AC group, 27 out of 76 participants were included in the measurement of copy numbers.

### Isolation of nuclear DNA and mitochondrial DNA

To compare the difference between n-cfDNA and mt-cfDNA in terms of promoting pro-inflammatory response, we independently isolated nDNA and mtDNA from murine liver tissue. The nuclear components were isolated using NE-PER Nuclear and Cytoplasmic Extraction Reagents (Thermo Fisher Scientific), and mitochondrial fractions were isolated by using the differential centrifugation method^14^. nDNA and mtDNA were fragmented on ice using an ultrasonicator (UD-211, TOMY SEIKO, Co., Ltd., Tokyo, Japan) at a power output of 3 for 2 × 30 s to simulate circulating n-cfDNA and mt-cfDNA^15^.

### Quantification of pro-inflammatory cytokines

To assess if total cfDNA, n-cfDNA, or mt-cfDNA could promote an inflammatory response, we applied these cfDNAs (cfDNA, 25 mg/mL, 250 ng/mL or 500 ng/mL; n-cfDNA, 500 ng/mL; mt-cfDNA, 500 ng/mL) to a murine macrophage cell line (J774.1). The dose of applying cfDNA was determined based on the hypothesis local cfDNA might be higher than that in systemic circulation. After 4 h of incubation with cfDNAs, RNA was extracted using RNeasy Mini Kit (QIAGEN). The expression of mRNA was calculated by using the 2^ΔΔ^ method with GAPDH as an internal control. Target pro-inflammatory cytokines consisted of IL-1β, IL-6, tumor necrosis factor-α (TNF-α), and monocyte chemoattractant protein-1 (MCP-1) (primer sequences are shown in S1).

### Evaluation of the involvement of TLR9 and importance of CpG methylation

To evaluate if mt-cfDNA was recognized through TLR9, J774.1 cells were pre-treated for 4 h with 2.5 µM of TLR9 inhibitor (ODN 2088) or the control (ODN 2088 control, Thermo Fisher Scientific), for the quantification of pro-inflammatory cytokines.

To assess if unmethylated CpG motif of mtDNA was essential in promoting pro-inflammatory response, we prepared both totally unmethylated mtDNA and fully methylated mtDNA. We designed 8 primer pairs covering whole mtDNA and obtained totally unmethylated mtDNA fragments using conventional PCR with Ex Taq DNA polymerase (TaKaRa). The mtDNA fragments were incubated with M.SssI, CpG Methyltransferase (New England Biolabs, Beverly, MA, USA), and S-adenosyl methionine at 37 °C for 1 h. Before the application to J774.1, both unmethylated and methylated mtDNA were fragmented by using an ultrasonicator, as mentioned earlier to simulate cf-mtDNA.

### Bisulfite sequence of heart and liver mtDNA

We performed whole mtDNA bisulfite sequencing by using a next generation sequencer with an originally modified protocol from the post bisulfite adaptor tagging method^16^. In brief, mitochondria were extracted from hearts and livers of mice, as mentioned above. EZ DNA Methylation-Gold kit (Zymo Research, Orange, CA, USA) was used for bisulfite treatment according to manufacturer’s instructions. The generation of barcoded libraries was carried out by using the Ion Plus Fragment Library Kit and Ion Xpress Barcode Adapters Kit (Thermo Fisher Scientific). Sequencing was conducted by using the Ion PGM with a 318 chip. The mapped reads with high mapping quality were used for extraction of methylated cytosines by using the Bismark methylation extractor^17^.

### Statistics

Continuous data were expressed as mean ± SEM or median [interquartile range], and categorical data as number and ratio, except for specifically described. An unpaired t-test, or Mann–Whitney U test was performed for continuous data, and the Chi-square test was for categorical data. Multiple group comparison was analyzed by ANOVA or Kruskal–Wallis test, followed by Tukey or Steel–Dwass tests, respectively. To investigate if cfDNA (total cfDNA, n-cfDNA, and mt-cfDNA) were useful as biomarkers for predicting AF incidence (sinus rhythm vs. PAF and PerAF) or AF progression (sinus rhythm and PAF vs. PerAF), we performed receiver operating characteristic (ROC) curve analysis using dataset excluding YC group. A *p* < 0.05 was considered statistically significant. Statistical analyses were performed using R software packages, version 3.2.2 (R Development Core Team, Vienna, Austria).

## Results

### Baseline characteristics of clinical samples

We analyzed 158 subjects, consisting of 104 controls and 54 patients with AF: YC (n = 28), AC (n = 76), PAF (n = 26), and PerAF (n = 28). The baseline characteristics of the subjects are summarized in Table 1. In comparison among AC, PAF, and PerAF, there were no significant differences in age, gender, or the histories of hypertension and diabetes, while we found significant differences in the history of dyslipidemia.

**Table 1:** Baseline characteristics of study subjects.

	Young control n = 28	Aged control n = 76	Paroxysmal AF n = 26	Persistent AF n = 28	P for 4 group	P for 3 group†
Age	25.1 (3.8)	66.2 (13.2)	67.7 (10.1)	67.4 (7.5)	< 0.001	0.797
Male, n (%)	14 (50)	53 (69.7)	16 (61.5)	23 (82.1)	0.066	0.239
Hypertension, n (%)	0 (0)	44 (57.9)	14 (53.8)	15 (53.6)	< 0.001	0.893
Diabetes, n (%)	0 (0)	18 (23.7)	3 (11.5)	4 (14.3)	0.039	0.301
Dyslipidemia, n (%)	0 (0)	37 (48.7)	10 (38.5)	3 (10.7)	< 0.001	0.002

Data were shown as numbers (%) (Male, Hypertension, Diabetes, and Dyslipidemia), mean (standard deviation) (Age). AF refers to atrial fibrillation.

^†^Comparison among aged control, paroxysmal AF and persistent AF.

### Elevation of cfDNA in AF patients

**Figure 1:**
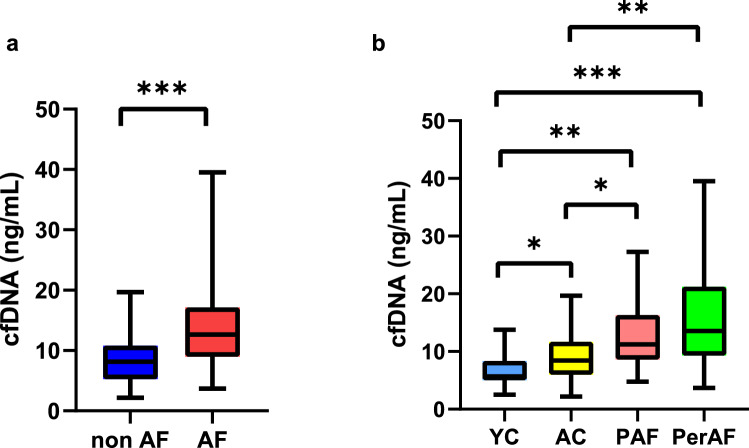
Elevation of total cfDNA in AF patients. (**a**) Comparison between non-AF and AF. (**b**) Comparison among the 4 subgroups. Mann–Whitney test was performed for a, Kruskal–Wallis test followed by post-hoc Steel–Dwass for b. *p < 0.05, **p < 0.01, ***p < 0.001.

The concentration of cfDNA was significantly higher in AF group than in non-AF groups (12.6 [9.0–17.1] vs. 8.1 ng/mL [5.3–10.8], *p* < 0.001) (Fig. 1a). The 4-group comparison showed that PerAF group had significantly higher cfDNA levels than YC (*p* < 0.001) and AC (*p* = 0.001) groups (13.6 [9.3–21.3], 5.7 [5.0–8.4], and 8.4 ng/mL [6.0–11.7] in PerAF, YC, and AC, respectively) (Fig. 1b). Likewise, PAF group had higher cfDNA levels (11.2 [8.6–16.3] ng/mL) than YC (*p* < 0.001) and AC (*p* = 0.013).

**Figure 2:**
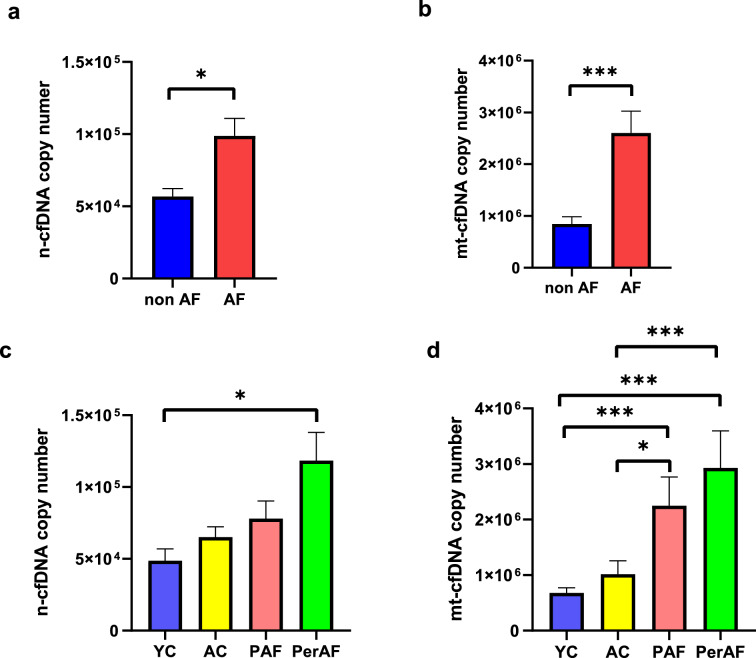
mt-cfDNA, rather than n-cfDNA, was increased in AF patients. Comparison between non-AF and AF for n-cfDNA (**a**) and mt-cfDNA (**b**). Comparison among the 4 subgroups for n-cfDNA (**c**) and m-cfDNA (**d**). Mann–Whitney test was performed for (**a**) and (**b**), Kruskal–Wallis test followed by post-hoc Steel–Dwass for (**c**) and (**d**). *p < 0.05, **p < 0.01, ***p < 0.001.

Next, we compared the absolute copy number of n-cfDNA and mt-cfDNA between AF and non-AF groups; AF group had significantly higher copy numbers in both n-cfDNA (*p* = 0.011) and mt-cfDNA (*p* < 0.001) than in non-AF group (Fig. 2a, b). In comparison of n-cfDNA level among the 4 groups, a significant difference was observed only between YC and PerAF groups (*p* = 0.015) (Fig. 2c). In contrast, both PAF and PerAF groups had statistically higher mt-cfDNA compared with YC and AC groups (*p* < 0.001 in YC vs. PAF, *p* = 0.017 in AC vs. PAF, *p* < 0.001 in YC vs. PerAF, *p* < 0.001 in AC vs. PerAF) (Fig. 2d). These findings indicate that the copy number of mt-cfDNA is potentially a better biomarker to identify AF, both in paroxysmal and persistent AF.

### Release of cfDNA from atrial cardiomyocytes under rapid stimulation

**Figure 3:**
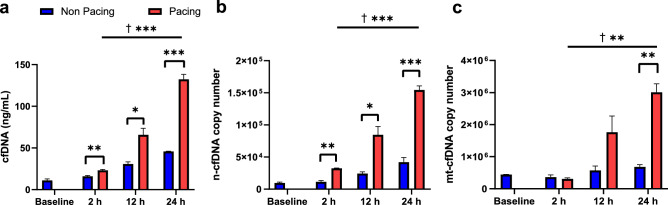
Total cfDNA, n-cfDNA, and mt-cfDNA in in vitro AF mimicking model. (**a**) Total cfDNA, (**b**) n-cfDNA copy number, (**c**) mt-cfDNA copy number in an in vitro tachycardia pacing model (n = 3 in each pacing duration). Absolute n-cfDNA and mt-cfDNA copy numbers were calculated using qPCR, using the created standard curves. Unpaired t test was performed. ANOVA test were followed by post-hoc Tukey for a in comparison among pacing groups (†). *p < 0.05, **p < 0.01, ***p < 0.001, † in comparison among pacing groups.

To confirm that cfDNA was released from atrial cardiomyocytes in the condition simulating tachycardia, we applied rapid pacing to HL-1 cells, and extracted cfDNA from the conditioned medium at several time points. The cfDNA level was significantly higher in the pacing group compared with the non-pacing group at each time point (15.97 ± 0.99 vs. 23.08 ± 1.05 (*p* = 0.008), 30.67 ± 2.60 vs. 65.67 ± 7.84 (*p* = 0.013), and 45.80 ± 0.28 vs. 132.33 ± 5.92 (*p* < 0.001) (ng/mL) at 2, 12, and 24 h of pacing, respectively) (Fig. 3a). The pacing stimulation for HL-1 induced higher level of cfDNA release from than that for NIH-3T3 cells at 24 h pacing duration (*p* = 0.024) (Supplementary Figure S1). To investigate if the cfDNA was released due to cell damage by pacing itself, we applied same rapid pacing to murine fibroblasts, NIH-3T3 cells. The cell death was evaluated by PI-staining. The ratio of PI-positive cells after 24 h pacing was lower in HL-1 cells than that in NIH-3T3 cells (9.3 ± 1.3% and 28.3 ± 1.5%, *p* < 0.001), implying that HL-1 cells might release cfDNA not only from dead cells, but by another mechanism from viable cells on pacing stimulation. Rapid pacing stimulation for HL-1 cells increased both n-cfDNA and mt-cfDNA copy number in a time-dependent manner (*p* = 0.002 and *p* = 0.008, respectively) (Fig. 3b, c). Compared with the non-pacing group, the copy number of n-cfDNA was higher at each pacing time point, and that of mt-cfDNA was higher at 24 h of pacing.

### Release of cfDNA in an in vivo murine atrial tachycardia model

**Figure 4:**
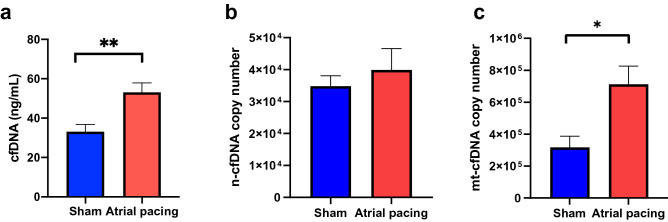
Total cfDNA, n-cfDNA, and mt-cfDNA in an in vivo AF mimicking model. (**a**) Total cfDNA, (**b**) n-cfDNA copy number, (**c**) mt-cfDNA copy number in an in vivo tachycardia pacing model (n = 7). Absolute n-cfDNA and mt-cfDNA copy number were calculated using qPCR using the created standard curves. * *p* < 0.05.

Next, we assessed the cfDNA level in an in vivo murine rapid atrial pacing model. After rapid pacing (1500 bpm) for 2 h, circulating blood was collected, and the cfDNA level was evaluated. cfDNA level was increased in the rapid pacing group compared with the sham group (53.11 ± 4.79 vs. 33.13 ± 3.67 (ng/mL), *p* = 0.007) (Fig. 4a). We also compared the copy number of n-cfDNA, showing that the copy number of n-cfDNA was similar between rapid pacing and sham groups (*p* = 0.901) (Fig. 4b). On the contrary, the copy number of mt-cfDNA was significantly higher in the atrial rapid pacing group than in sham group (*p* = 0.011) (Fig. 4c).

### cfDNA promotes IL-1β and lL-6 expression in macrophages

**Figure 5:**
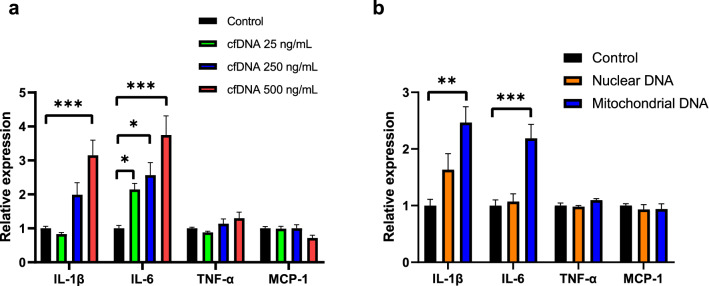
mt-cfDNA, but not n-cfDNA, promoted IL-1β and IL-6 expression in macrophages. (**a**) Total cfDNA (25 ng/mL, 250 ng/mL or 500 ng/mL) (n = 4–8), (**b**) n-cfDNA (500 ng/mL), and mt-cfDNA (500 ng/mL) (n = 5–8) were applied to J774.1, then pro-inflammatory cytokines expression was analyzed using qPCR. Nuclear and mitochondrial DNA were independently extracted and fragmented to simulate n-cfDNA and mt-cfDNA. ANOVA followed by post-hoc Tukey was performed. * *p* < 0.05, ** *p* < 0.01, *** *p* < 0.001.

To examine if cfDNA released from cardiomyocytes promoted production of pro-inflammatory cytokines, we applied cfDNA to macrophages (J774.1). We performed rapid pacing of HL-1 cells for 24 h and extracted cfDNA from the conditioned medium. The cfDNA released from HL-1 significantly promoted the expression of IL-1β in a dose-dependent fashion (fold change: 0.83 (*p* = 0.944), 1.99 (*p* = 0.092) and 3.15 (*p* < 0.001) in 25, 250 and 500 ng/mL, respectively). The expression of IL-6 also increased dose-dependently (fold change: 2.14 (*p* = 0.025), 2.56 (*p* = 0.023) and 3.75 (*p* < 0.001) in 25, 250 and 500 ng/mL, respectively). However, the level of TNF-α and that of MCP-1 were not changed by application of cfDNA (Fig. 5a).

To determine the difference of n-cfDNA and mt-cfDNA in this pro-inflammatory effect, we independently applied fragmented n-cfDNA and mt-cfDNA to J774.1 cells. Of note, IL-1β and IL-6 expressions were significantly increased by the application of mt-cfDNA (fold change, 2.47 (*p* = 0.001) and 2.19 (*p* < 0.001) for IL-1β and IL-6, respectively), but not by application of n-cfDNA (Fig. 5b). Consistent with the result of total cfDNA, the level of TNF-α and MCP-1 did not change by the application of mt-cfDNA and n-cfDNA.

### Unmethylated mt-cfDNA activates inflammatory responses in macrophages via TLR-9

**Figure 6:**
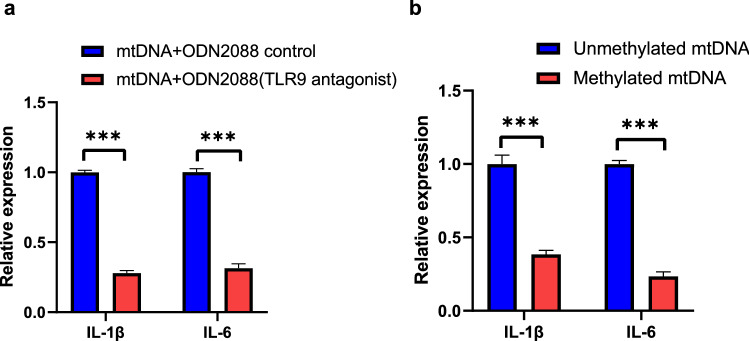
Sparsely methylated mt-cfDNA induced IL-1β and IL-6 expression in macrophages via TLR9. (**a**) Comparison of IL-1β and IL-6 expression levels between TLR9 inhibitor vs. ODN control under mitochondrial DNA exposure (500 ng/mL) (n = 4–6). (**b**) Comparison of IL-1β and IL-6 expression levels between fully methylated mitochondrial DNA (500 ng/mL) versus totally unmethylated mitochondrial DNA (500 ng/mL) (n = 6). Unpaired t test was performed for (**a**) and (**b**) *** *p* < 0.001.

We next examined if these pro-inflammatory responses by mt-cfDNA were activated via TLR-9. Administration of TLR9 inhibitor (ODN2088) significantly suppressed the induction of IL-1β and IL-6 expression, compared with ODN 2088 control (Fig. 6a).

Since it was reported that TLR9 recognized unmethylated CpG^12^, we evaluated the ratio of unmethylated CpG in mt-cfDNA using bisulfite sequence with next generation sequencers. The ratio of unmethylated cytosines in mtDNA obtained from murine hearts was 99.6%, and that from liver was 99.7%. These findings indicated that mtDNA was highly unmethylated, regardless of organs. Then, we applied mt-cfDNA to macrophage cell lines with or without treatment with methyltransferase. The application of methyltransferase to mt-cfDNA significantly reduced the expression of IL-1β and IL-6 (Fig. 6b).

### Usefulness of cfDNA as a biomarker for AF

**Figure 7:**
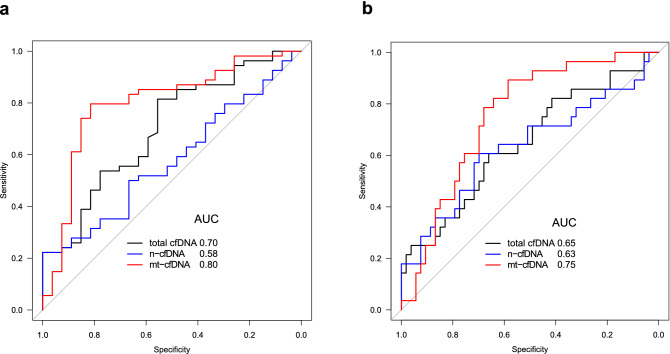
Receiver operating curve (ROC) curve analysis for AF incidence and progression. ROC analysis for AF incidence (**a**) (non-AF vs. AF), and for AF progression (**b**) (non-AF and paroxysmal AF vs. persistent AF) using total cfDNA, n-cfDNA, and mt-cfDNA. AUC, area under the curve.

We performed ROC curve analysis to determine the diagnostic utility of total cfDNA, n-cfDNA, and mt-cfDNA for the prediction of AF incidence and that of AF progression from paroxysmal AF to persistent AF. Since YC group had different background, we excluded it from this analysis. Figure 7a shows the ROC curve of total cfDNA, n-cfDNA, and mt-cfDNA for AF incidence. The area under the curve (AUC) was 0.70 (95% confidence interval [CI], 0.58–0.82) for total cfDNA, 0.58 (95% CI 0.45–0.71) for n-cfDNA, and 0.80 (95% CI 0.69–0.91) for mt-cfDNA, respectively. Since the cfDNA level was higher in PerAF than in PAF, we evaluated if cfDNA predicted the progression of AF. The AUC was 0.65 (95% CI 0.52–0.78) in total cfDNA, 0.63 (95% CI 0.50–0.77) in n-cfDNA, and 0.75 (95% CI 0.65–0.86) in mt-cfDNA (Fig. 7b). Thus, mt-cfDNA appeared to be the most promising biomarker to predict the incidence of AF and the progression of AF.

## Discussion

In this study, we found that a higher cfDNA level was associated with AF in both clinical and experimental settings. Especially, the copy number of mt-cfDNA was more strongly associated with AF than n-cfDNA. To the best of our knowledge, this is the first report documenting the association between cfDNA and pathophysiology of AF. We also clarified the functional role of mt-cfDNA as promoting pro-inflammatory cytokines, such as IL-1β and IL-6 in macrophages. The unmethylated CpG in mt-cfDNA was essential for this pro-inflammatory response, and TLR9 was involved in this response. It has been well known that the sterile systemic inflammation was accompanied by AF^5^. The current study indicated that sparsely methylated mt-cfDNA might play a critical role for this inflammatory response in AF.

We observed that AF patients had a higher cfDNA level compared with age-matched non-AF subjects. This finding was consistent with those in vivo- and in vitro-AF mimicking models. The pacing stimulation on atrial cardiomyocytes caused cfDNA release from cells, and pacing stimulation on atrial tissue in mouse caused elevation of cfDNA in mice. Several previous studies mentioned that cfDNA worked as DAMPs in inflammatory disorders^9,18^. In the cardiovascular field, patients with myocardial infarction were known to have a higher cfDNA level in plasma than healthy subjects^11^. Our study showed that cfDNA in AF settings induced the systemic inflammation, possibly explaining how the AF itself induces systemic inflammation, the mechanism of AF begets AF, and one of the mechanisms underlying progression to persistent AF in about 9 to up to 30% of paroxysmal AF patients in a year^19^.

cfDNA could be divided into 2 types by its source: mt-cfDNA and n-cfDNA. We demonstrated that mt-cfDNA rather than n-cfDNA was strongly related to AF in clinical samples. Consistently, we also showed that rapid pacing at right atrium increased mt-cfDNA, but not n-cfDNA level in the murine model. A previous study showed the increase of mitochondria number in AF heart using the goat chronic AF model^20^, which may help explain why mt-cfDNA rather than n-cfDNA level increased in the patients with AF and in the rapid pacing model. On the contrary, Soltesz et al. has recently reported that mtDNA copy number in peripheral blood did not differ in the patients with AF^21^. Further studies are needed to elucidate which factor or condition regarding AF may affect mt-cfDNA levels.

Our study revealed that cfDNA released from atrial cardiomyocytes promotes IL-1β and IL-6 expression in macrophages. In detail, mt-cfDNA, but not n-cfDNA, activated macrophages for inducing pro-inflammatory cytokines via TLR9. In several previous studies, cfDNA^10,22^ and mtDNA^15,23,24^ levels have been reported to be associated with inflammatory cytokines. Our study differs from others in the fact that we directly compared the inflammatory response between n-cfDNA and mt-cfDNA. To date, several receptors and molecular pathways are reported in innate immune response: TLR9, NLRP3 (mediates the secretion of IL-1β and IL-18), and stimulator of interferon genes (STING) signaling (stimulates the expression of type I interferon)^25^. In agreement with our findings, prior studies showed that mtDNA levels were recognized through TLR-9 as DAMPs, subsequently activated the nuclear factor kappa B (NFκB) signaling, and increased the expression of IL-1β and IL-6^26^. On the other hand, Yao et al. has documented that NLRP3 inflammasome activity was increased in the atrial cardiomyocytes of the patients with AF, and inhibition of NLRP3 reduced AF inducibility^27^. Of note, TLR9 has been reported as the key upstream molecule in NLRP3 inflammasome activation^28^. Further studies are needed to evaluate whether cf-mtDNA released from cardiomyocytes could activate NLRP3 inflammasome directly or indirectly, and subsequently promote AF pathogenesis.

cf-mtDNA has several unique features: mtDNA is a small, double-stranded circular molecule; hundreds to thousands of mtDNA copies are present in each cell, and mtDNA is methylated in a different way from nuclear DNA. These characteristics make it more ‘foreign’ than ‘self’ DNA^25^. We especially focused on the influence of CpG methylation of mtDNA, because TLR-9 preferentially recognizes unmethylated CpG. According to our direct comparison between fully unmethylated cf-mtDNA and methylated cf-mtDNA, unmethylated CpG accounted for about 70% of IL-1β and IL-6 induction by mt-cfDNA. Interestingly, the mechanism of methylation in mtDNA remains highly debated. Several groups demonstrated the presence of 5-methyl cytosine in mtDNA using different approaches^29,30^, however, others showed the opposite evidence that mtDNA was not methylated^31–33^. In the current study, we observed that the mtDNA in murine heart was barely methylated (0.4%) using whole mtDNA bisulfite sequencing. This finding supports the idea that mt-cfDNA released from the heart could cause systemic inflammation.

mt-cfDNA may be a potential novel biomarker for the prediction of AF incidence and progression of AF. Several candidate biomarkers for detection of AF have been proposed, such as N-terminal prohormone of brain natriuretic peptide (NT-proBNP)^34^ and brain natriuretic peptide (BNP)^35^ reflecting atrial strain, and Galectin-3 correlating with cardiac fibrosis^36^. We recently reported that several circulating microRNAs were also useful^37^. Notably, CRP also has been reported as an independent predicter for AF^38^. The current study revealed that mt-cfDNA promoted IL-1β and IL-6 expression in macrophages which led to CRP induction, suggesting that mt-cfDNA could be an upstream factor in the inflammatory response in AF. Further studies are needed to investigate whether the addition of mt-cfDNA to conventional risk factors could improve the prediction accuracy for AF.

Our study has several limitations. First, the number of clinical subjects in each group was relatively small, and the established risk factors for AF, such as body mass index or status of alcohol consumption were not available. Further, a large and prospective study is required to confirm the association between mt-cfDNA and AF incidence and progression. Second, the blood was collected without paying attention to the heart rhythm at the time point of the puncture. Especially in patients with PAF, this factor might affect the accuracy of the prediction. Third, we have not assessed the confounding factors influencing the level of cfDNA. Several factors may affect the quantification of cfDNA, including blood pressure, heart rate, meal, and diurnal variation. Not only cardiomyocyte, but other cells including fibroblast or endothelial cells in the heart might be a source of cfDNA under AF. Although AF is associated with an increased risk of systemic comorbidities and complications^1^, cfDNA could be theoretically affected by systemic whole cells’ condition. Recently, FAM101A has been reported as a biomarker to quantify cardiomyocyte-specific DNA in cfDNA^39,40^. Future direct comparison mt-cfDNA and FAM101A will need to explore the useful prediction model for AF incidence and progression by using cfDNA. Fourth, we performed very rapid pacing stimulation in our murine experimental model, which might partly evoke cell death, resulting in the elevation of cfDNA. Further study using large animals may be needed to perform atrial pacing like human AF.

## Conclusion

In conclusion, the patients with AF showed higher levels of cfDNA, in particular mt-cfDNA. Sparsely methylated mt-cfDNA promoted IL-1β and IL-6 expression in macrophages via TLR9. Sparsely methylated mt-cfDNA may contribute to the sterile systemic inflammation accompanied by AF, indicating that mt-cfDNA may potentially be a novel therapeutic target and biomarker for AF.

## Supplementary Information

Supplementary Information.

## Data Availability

All data associated with this study are in the paper or the Supplementary Materials.
