# Supplementary material for: Sparsely methylated mitochondrial cell free DNA released from cardiomyocytes contributes to systemic inflammatory response accompanied by atrial fibrillation
Source: Sci Rep. 2021 Mar 18;11:5837. doi: 10.1038/s41598-021-85204-7 (PMC7973420; doi:10.1038/s41598-021-85204-7)
Supplement: Supplementary file 1 — Supplementary Information. [file 41598_2021_85204_MOESM1_ESM.pdf]

Supplementary information

Sparsely Methylated Mitochondrial Cell free DNA Released from Cardiomyocytes Contributes to Systemic Inflammatory Response Accompanied by Atrial Fibrillation

Authors:

Masahiro Yamazoe[1,2], MD, Ph.D., Tetsuo Sasano[2,3*], MD, Ph.D., Kensuke Ihara[1], MD, Ph.D., Kentaro Takahashi[1], MD, Ph.D., Wakana Nakamura[2], Naomi Takahashi[2], Hiroaki Komuro[2], Satomi Hamada[2], and Tetsushi Furukawa[1], MD, Ph.D.

1）Department of Bio-informational Pharmacology, Medical Research Institute, Tokyo Medical and Dental University, Tokyo, Japan

2) Department of Cardiovascular Physiology, Tokyo Medical and Dental University, Tokyo, Japan

3) Department of Cardiovascular Medicine, Tokyo Medical and Dental University, Tokyo, Japan

Supplementary Table S1

Name	Forward	Reverse
GAPDH (human for copy number)	CCCCACACACATGCACTTACC	CCTAGTCCCAGGGCTTTGATT
NADH (human for copy number)	ATACCCATGGCCAACCTCCT	GGGCCTTTGCGTAGTTGTAT
GAPDH (mouse for copy number)	AACTCCTCATGGGTCTGTAGTGA	GTACGTGCATAGCTGATGGCTG
NADH (mouse for copy number)	CTCCTCAGTTAGCCACATAGCA	GTGAGGCCATGTGCGATTAT
GAPDH (mouse for internal control)	TGTGATGGGTGTGAACCACGAGAA	GAGCCCTTCCACAATGCCAAAGTT
IL-1β	CTGGTGTGTGACGTTCCCATTA	CCGACAGCACGAGGCTTT
IL-6	ACAACCACGGCCTTCCCTACTT	CACGATTTCCCAGAGAACATGTG
TNFα	ACCCTCACACTCAGATCATCTTC	TGGTGGTTTGCTACGACGT
MCP-1	TCACCTGCTGCTACTCATTCACCA	TACAGCTTCTTTGGGACACCTGCT
mitochondria_1	AAGGTTTGGTCCTGGCCTTA	AGAGACAGTTGGACCCTCGT
mitochondria_2	CCCAACACCGGAATGCCTAA	CAACGTTTTCGGGGTATGGG
mitochondria_3	ATCCTCCTGGCCATCGTACT	CTCCGTGTAGGGTTGCAAGT
mitochondria_4	ATCTGTTCTGATTCTTTGGGCAC	TACGGCTCCAGCTCATAGTG
mitochondria_5	TGCAGGATTCTTCTGAGCGT	TGGATCCGTTCGTAGTTGGA
mitochondria_6	CCAACTACGAACGGATCCACA	ATGGTATTCCTGTGAGGGCG
mitochondria_7	CCTCGCCCTCACAGGAATAC	AGCTCCTTCTTCTTGATGTCTTGA
mitochondria_8	AGCACCCAAAGCTGGTATTC	AAGTACCGCCAAGTCCTTTGA

Figure S1

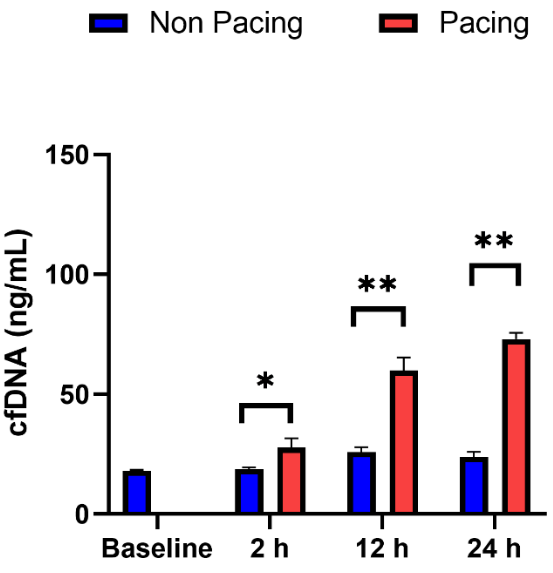

Figure S1: **Total cfDNA in *in vitro* pacing to NIH-3T3 cell.**

Total cfDNA in an *in vitro* tachycardia pacing to NIH-3T3 cell (n = 6 in each pacing duration).

Unpaired t test was performed. * $p < 0.05$, ** $p < 0.01$.
